# Comments on Melis *et al*. The Effects of the Urban Built Environment on Mental Health: A Cohort Study in a Large Northern Italian City. *Int. J. Environ. Res. Public Health*, 2015, *12*, 14898–14915

**DOI:** 10.3390/ijerph13030250

**Published:** 2016-02-24

**Authors:** Yan Kestens, Basile Chaix, Martine Shareck, Julie Vallée

**Affiliations:** 1 Research Theme of Risks, Prevention and Health Promotion, Montreal University Research Center (CRCHUM), 850, Rue St.-Denis, Montréal, QC H2X 0A9, Canada; 2 Department of Social and Preventive Medicine, École de Santé Publique de l’Université de Montréal (ESPUM), 7101, Rue du Parc, Montréal, QC H3N 1X9, Canada; 3 Inserm, UMR-S 1136, Pierre Louis Institute of Epidemiology and Public Health, Nemesis research team, 27 Rue Chaligny, 75571 Paris, France; basile.chaix@upmc.fr; 4 Department of Social and Environmental Health Research, London School of Hygiene and Tropical Medicine, 15-17 Tavistock Place, London WC1H 9SH, UK; martine.shareck@lshtm.ac.uk; 5 Centre National de la Recherche Scientifique (CNRS), UMR Géographie-Cités, 13 Rue du Four, 75006 Paris, France; julie.vallee@parisgeo.cnrs.fr

In a recent paper by Melis and colleagues [1], exposure to certain built environment characteristics—urban density and accessibility to public transit—is found to be related to mental health, even more so among women, the elderly, and the residentially stable (interactions between built environment and individual characteristics in relation to mental health have unfortunately not been tested statistically, which could have strengthened their demonstration). The authors argue that this may be because these groups spend more time in their neighbourhoods [1]. Other studies have associated the length of time spent in home neighborhoods with stronger links to health outcomes, e.g., Chum, A., *et al*. [2], or mentioned this hypothesis [3,4]. One interpretation of such findings may be, as the authors suggest, that some sort of dose-response relationship is at play—the longer the exposure, the stronger the influence. However, this interpretation also reveals limitations in how environmental exposure is being measured, and, more globally, in how little actual processes linking exposure to health are being documented and understood, *i.e.*, “how environments actually get under the skin”.

Regarding measures of environmental exposure, a large body of research is still focusing on residential environments only. However, following early calls to avoid local [5] or residential traps [6], and acknowledging studies that showed differences in exposures between home and non-residential destinations [7,8,9,10], activity space approaches attempting to account for daily mobility and multiple exposures are increasingly being used. Such approaches try to account for non-residential exposures, by using mobility data from surveys of regular destinations [11,12] or from GPS tracking [13], by integrating information on time spent within and outside the residential neighbourhood, or by using measures of concentration of activities within/outside the residential neighbourhood [3,4]. As people move around to conduct their daily activities, they may visit a number of non-residential places. Consequently, they are exposed to sometimes contrasting environments while spending time at these locations or during trips along routes. Ignoring such non-residential exposures is problematic both because there are reasons to believe that they may also play a role (unless there is a specific *a priori* hypothesis that only residential exposure has an influence) and because doing so may translate into systematic bias, or misclassification.

Our first argument is to reiterate previous statements that ignoring non-residential environments provides a partial picture of actual exposure. This means that providing comprehensive measures of exposure implies accounting for the various locations and corresponding contrasting exposures that people experience. Doing so does not mean, however, that all locations are of the same importance. Because precise processes linking environments to health remain largely unknown, more comprehensive data collection on daily mobilities, activities and conditions of environmental exposure is needed. Beyond the multiple exposures themselves, other dimensions, yet undocumented, may indeed play a role in the dose-response relationship linking exposure to health. Exposure in some destinations may play a larger role than in others because of the nature of activities that are conducted at these locations—e.g., workplace *vs.* leisure place—because of the influence of time of day or day of the week, because social dimensions, such as co-presence or absence of social network members modify how environments are perceived, or because other psychosocial dimensions render ‘that place at that moment’ specifically important. In other words, not only should multiple destinations be considered to improve specificity in exposure measures, but there should also be a much richer documentation of the conditions under which these exposures are being experienced.

Our second argument presents the possibility of systematic bias, as it appears that misclassification in exposure, or the difference between actual exposures experienced throughout daily mobilities and exposure measured in the residential environments only, should be smaller among those who spend more time in their neighbourhood, and consequently larger if they spend more time at other locations. The difference will further increase along with increasingly contrasting characteristics of these other locations compared to the home environment. It is important to state here that both dimensions—*i.e.*, the time spent within or outside the residential neighborhood, itself linked to daily mobility patterns, and the range of difference between residential and non-residential environments may vary according to individual profiles. This means that magnitudes of misclassification in measures of exposure based on home environments only may be related to other traits such as age, socio-economic status, occupation, or gender [14,15], resulting in biased effect estimates.

These two arguments call for a change in how research on place effects is conducted. We provide a number of recommendations to incorporate such change. First, spatial data on daily mobility should be collected more often and more systematically to allow specific estimates of exposures in multiple destinations and along routes. Solutions to collect such data include map-based questionnaires, GPS tracking, or other surveys about where people spend time. Second, complementary information about ‘what happens where, when, with whom and why’ is needed to improve our understanding of the very processes linking environments to health. This is especially true when conscious or even unconscious cognitive processes such as chronic stress and allostatic load, feelings of safety, or perceptions of environmental cues that shape behaviour are involved. In short, exposure to environments needs to be further contextualised and individualized. Accounting for complementary conditions such as activities being conducted, time of day, presence and relation with peers, or variations in individuals’ feelings and transient mind states will contribute to an improved understanding of how environments get under the skin. Various tools, including ecological momentary assessment, socio-spatial questionnaires, or individualised assessments through go-along methods or ethnographic geographies, can be used to collect such information. Of course, with an increasing number of variables to be considered, and possible explorations of various moderation hypotheses, issues of power may arise. 

Regarding this point, we further believe that a stronger consideration of not only environmental exposure conditions, but rather of changes or variations thereof will help increase power and identify meaningful associations. If environmental conditions do in fact play a role, tracking their changes and linking those changes to variations in behaviour or health will help reveal significant pathways. Relating changes in exposure to changes in health not only increases the strength of causal proof, it also increases the power for detection. Consequently, we further argue that more efforts should be invested to assess change in environmental conditions in relation to change in health states. Such changes may be observed within days, through micro-level and continuous monitoring of variations in environmental exposure and health across space and time within individuals, or within longer periods, with changes in environments possibly documented through space-time geographic information systems, and changes in health monitored through systematic longitudinal designs. 

More should be done to document environmental change, including the development of data platforms with systematic and continuous monitoring of environmental status, and further integration of algorithms to produce indicators of environmental change. Furthermore, because changes in the environment often result from planned decisions, an improved monitoring of urban interventions and modifications of built environments is warranted. With cities increasingly producing and relying on sensor networks, and people increasingly generating individualised spatio-temporal information on mobilities and health behaviour, new data and computing capabilities can help our field of research understand how people and places interact. With new possibilities also come new challenges, and increasingly multidisciplinary perspectives will be required to fully address such complexities. The development of larger cohorts with detailed and continuous monitoring of behaviour and health, including daily mobility and social contacts, should be promoted. Novel tools, such as web-based questionnaires, or wearable sensors, including mobile phone sensing, can help to achieve this goal. While we realise this commentary’s recommendations go well beyond the paper by Melis *et al.*, tackling built environment effects on mental health, we acknowledge the authors’ important research question and hope this commentary can help our field move forward.
